# Capacity building of health care professionals to perform interprofessional management of non-communicable diseases in primary care – experiences from Ukraine

**DOI:** 10.1186/s12913-021-06068-1

**Published:** 2021-01-27

**Authors:** Tiina Laatikainen, Anastasiya Dumcheva, Tetiana Kiriazova, Oleksandr Zeziulin, Laura Inglin, Dylan Collins, Jill Farrington

**Affiliations:** 1 Institute of Public Health and Clinical Nutrition, University of Eastern Finland, Kuopio, Finland; 2 Finnish Institute for Health and Welfare, Mannerheimintie 166, 00300 Helsinki, Finland; 3 Joint municipal authority for North Karelia health and social services (Siun sote), Joensuu, Finland; 4 Ministry of Health of Ukraine, Kyiv, Ukraine; 5 Ukrainian Institute on Public Health Policy, Kyiv, Ukraine; 6 University of British Columbia, Vancouver, Canada; 7 World Health Organization Regional Office for Europe, Copenhagen, Denmark

**Keywords:** Primary health care, Non-communicable diseases, Intervention, Training, Disease management, Total risk assessment, Treatment outcomes, Quality improvement

## Abstract

**Background:**

Non-communicable diseases are leading causes of death and disability across the world. Countries with the highest non-communicable disease (NCD) burden in the WHO European Region are often those that have some of the greatest health system challenges for achieving good outcomes in prevention and care. The aim of this study was to evaluate the effect of an interprofessional capacity building intervention carried out in Ukraine to improve the management non-communicable diseases in primary health care.

**Methods:**

A mixed-methods evaluation study was performed in 2018 to analyse the effect of a capacity building intervention carried out for over 10,000 primary care professionals in Ukraine in 2018. Quantitative data were collected from primary health care records of intervention and control areas preceding the intervention and 1.5 to 2 years after the intervention. Altogether 2798 patient records before and 2795 after the intervention were reviewed. In control areas, 1202 patient records were reviewed. Qualitative data were collected carrying out focus group interviews for health professionals, clinic managers and patients. Also, observations of clinical practice and patient pathways were performed.

**Results:**

The capacity building intervention improved the capacity of professionals in detection and management of non-communicable disease risk factors. Significant improvement was seen in detection rates of both behavioural and biological risk factors and in medication prescription rates in the intervention areas. However, almost similar improvement in prescription rates was also observed in control clinics. Improvements in control of blood pressure, blood glucose and cholesterol were not seen during the evaluated implementation period. Qualitative analyses highlighted the improved knowledge and skills but challenges in changing the current practice.

**Conclusions:**

A large scale capacity building intervention improved primary health care professionals’ knowledge, skills and clinical practice on NCD risk detection and reduction. We were not able to detect improvements in treatment outcomes - at least within 1.5 to 2 years follow-up. Improvement of treatment outcomes would most likely need more comprehensive systems change.

**Supplementary Information:**

The online version contains supplementary material available at 10.1186/s12913-021-06068-1.

## Introduction

Noncommunicable diseases (NCDs) are leading causes of death and disability across the world, and an obstacle to economic development. Ukraine is a lower-middle income country in the European Region of WHO and in 2016, NCDs were estimated to account for 91% of its deaths [1]. The probability of dying between the ages 30 and 70 years from any of four major NCDs (cardiovascular diseases, cancer, diabetes mellitus, chronic respiratory diseases) in Ukraine in 2016 was amongst the highest for countries in the WHO European Region and twice as high for men (35.1%) as for women (16.2%) [2]. Smoking levels and harmful consumption of alcohol are high, particularly amongst men [3, 4]. Over a quarter of the adult population has raised blood pressure (SBP ≥ 140 and/or DBP ≥ 90 mmHg or is currently on medication for raised BP) [5].

Countries with the highest cardiovascular disease (CVD) burden in the WHO (World Health Organization) European Region are often those that have some of the greatest health system challenges for achieving good NCD outcomes [6]. Ukraine has been undertaking a major transformation of its health care system towards universal health coverage since 2015. The initial focus on health financing reform and building health information systems has led to introduction of new financing mechanisms, launch of an e-health system, and strengthening of primary care including reimbursement of selected medicines [7, 8].

WHO has identified a set of effective interventions for NCD prevention and control and has developed tools to assist in their implementation [9]. One such tool is the Package of essential NCD interventions (WHO PEN) which includes simple clinical protocols for integrated prevention and management of CVD and diabetes in primary health care (PHC) in low-resource settings [10].

The aim of this study is to report the results of a pilot project to implement WHO PEN related activities in Ukraine, using retrospective mixed-method effect evaluation after implementation, comparing the PHC clinics in the pilot and control regions.

## Methods

### Intervention description

The intervention focused on strengthening the knowledge and capacity for prevention and control of NCDs in clinical settings. One of the key activities was the training of PHC workers on the integrated management of hypertension and diabetes following the principles of the WHO Package of Essential Noncommunicable diseases interventions (WHO PEN) for PHC. The training was delivered as a 2-day course by 52 regional trainers in 7 pilot sites of the project (Ivano-Frankivsk, Lviv, Vinnitsa, Poltava, Dnipropetrovsk, and Kharkiv regions, and Kyiv city) between October 2016 and June 2018. Professionals were selected quasi-randomly into the interventions. At the first stage, to ensure equal representation of different rayons, each region was divided into sub-regional districts consisting of several rayons. Regional health authorities were randomly selecting health professionals from all PHC clinics providing services in these sub-regions and ordering them to attend the training. As a result, the training course was attended by 10,804 PHC professionals, and for example 55% of all doctors providing services in the seven pilot regions attended the training.

Other elements of the intervention comprised the provision of the WHO PEN protocols for ‘Integrated Management of Diabetes and Hypertension’ and ‘Health Education and Counseling on Healthy Behaviours’ adapted for Ukraine, clinical decision support tools, and tape measures for all participants of the training sessions. Training in use of these was an integral part of the cascade training provided. WHO also worked with the health authorities of the participating regions and Kyiv city to strengthen their broader governance/leadership role in NCD prevention; this element is described further elsewhere [11].

### Data collection

The effect of the intervention was evaluated using a mixed-methods model including both quantitative and qualitative data collection as well as clinic facility and patient pathway observations (Table 1).

**Table 1:** Summary table of key information of the three complementary data sources

Data	Key aspects	Data collection & data collection tool	Data collection period	1440 patients from control clinics (1202 baseline; 1202 at follow-up)
Quantitative patient record data	Changes in risk assessment and treatment practices and outcomes	Retrospective data collection from paper-based patient records in intervention and control clinics for a two-year period for baseline (1 Jan 2015 to 31 Dec 2016) and follow-up (1 Jan 2017 to 31 Dec 2018), utilizing a standardized data collection form (filled in online).	25 Jan 2019 to 8 Apr 2019	3360 patients from intervention clinics (2798 baseline; 2795 follow-up) 1440 patients from control clinics (1202 baseline; 1202 at follow-up)
Qualitative interview data	Professionals’ opinions on the effects of training in changing the practice and related possibilities and challenges	Focus group interviews (FGI) including managers of primary health care clinics, doctors, nurses, feldshers, and patients of the family doctors from intervention clinics, following a semi-structured interview scheme based on predefined themes.	20 Dec 2018 to 27 Feb 2019	A total of 15 FGI with workforce from intervention clinics: 3 FGIs with managers (*n* = 16) 3 FGIs with family doctors (*n* = 18) 3 FGIs with nurses (*n* = 22) 3 FGIs with feldshers (*n* = 18) 3 FGIs with patients (*n* = 25)
Observation data	Organizational changes in clinics, availability of tools and equipment, and changes in the division of tasks	Clinic facility and patient pathway observations in intervention and control clinics following a protocol and a structured observation scheme	25 Jan 2019 to 8 Apr 2019	112 observations in intervention clinics; 39 in control clinics One patient pathway observation (if possible) per clinic during the quantitative data collection

Abbreviations: *FGI* focus group interview

Quantitative data for the assessment of the intervention effect was collected from seven intervention regions (Ivano-Frankivsk Lviv, Vinnitsa, Poltava, Kharkiv, Dnipropetrovsk and Kyiv city) and from three control regions (Ternopil, Sumy, and Cherkasy) for the pre-training period (baseline) and post-training period (follow-up). A standardized online data collection tool (See Supplementary Table 1) was utilized to collect retrospectively from paper-based patient records. The data collection tool has been successfully used in prior studies [12–14].

Post-training data was collected for patients of doctors trained between 1st February 2017 and 30th June 2017 to guarantee that until the end of 2018 the professionals had had time to change their practices after training. In order to ensure that the implementation period for each professional was at least 1.5 years after training, post-training data was collected searching patient records 2 years backwards from the end of the year 2018 and pre-training data searching patient records 2 years backwards from the end of the year 2016. The data collection was done between 25 January 2019 and 8 April 2019.

The aim was to assess for both periods 400 patients per region to obtain a number of observations allowing analysis of key indicators by region. An equal number of trained doctors was sampled from each region (*n* = 20). Altogether 140 doctors were sampled randomly from intervention regions and 60 from control regions. The data was collected from patient records of those patients who according to the Ukrainian protocol are eligible for total cardiovascular risk assessment: men 40 years of age or older and women 50 years of age or older. In intervention regions, the patients also needed to be clients of a doctor trained in the project. For the post-training data collection, the first 12 eligible male patients (born 1976 or earlier) and first 12 eligible female patients (born 1966 or earlier) were selected from the visit record of each doctor from year 2018. Selecting altogether 24 patients per doctor allowed 20% drop-out from the final sample in situations where patient records for example were not found or patients kept those at home. Similarly, for the pre-training data collection, the first 12 eligible male patients (born 1974 or earlier) and the first 12 eligible female patients (born 1964 or earlier) were selected from the visit record of each doctor from year 2016. The intended sample size was 3360 patients from intervention regions and 1440 from control regions at both baseline and follow-up timepoints. However, not all the patient records were available and ultimately from the intervention areas, 2798 patient records at baseline and 2795 at follow-up were reviewed. In control areas, 1202 patient records were reviewed for both baseline and follow-up.

The qualitative data was collected through face-to-face, voice-recorded, 1.5–2 h lasting focus group interviews between 20 Dec 2018 and 27 Feb. The semi-structured interview scheme followed the topics presented in Table 2 (See also Supplementary Table 2). In total, 15 focus group interviews were conducted in three regions, of which three focus group interviews were conducted with managers (*n* = 16), three with doctors (*n* = 18), three with nurses (*n* = 22), three with feldshers (*n* = 18) and three with patents of trained family doctors (*n* = 25).

**Table 2:** Focus group interview themes

Clinic managers	
Describe and discuss the role that managers played in implementing the project.	
Describe and discuss the results or outcomes of the implementation.	
Describe and discuss how the role of nurses and/or feldshers changed?	
Describe and discuss how local protocols, guidelines, or practice recommendations were developed after training to support clinical decision making.	
Describe and discuss how patients are contacted or reminded to come to their follow-up medical appointment (“recall systems”)	
Describe and discuss any recommendations you would give to other clinics who want to implement the project.	
Doctors	
Describe and discuss what you learned from the training workshop	
Describe and discuss if and how the division of tasks of doctors, feldshers and nurses changed after the training?	
Describe and discuss the support you had from your clinic (e.g. managers) to make implement the changes you learned in the training	
Feldshers	
Describe and discuss what you learned from the training workshop	
Describe and discuss if and how the division of tasks of doctors, feldshers and nurses changed after the training?	
Describe and discuss the support you had from your clinic (e.g. managers) to make implement the changes you learned in the training	
Describe and discuss how your confidence in dealing with patients changed?	
Nurses	
Describe and discuss what you learned from the training workshop	
Describe and discuss if and how the division of tasks of doctors, feldshers and nurses changed after the training?	
Describe and discuss the support you had from your clinic (e.g. managers) to make implement the changes you learned in the training	
Describe and discuss how your confidence in dealing with patients changed?	
Patients	
Describe and discuss any changes in the care you received during the last year	
Describe and discuss how your blood pressure, blood sugar, waist circumference, and/or weight have been measured	
Describe and discuss if and how you have been asked about your smoking, alcohol use, diet, physical activity?	
Describe and discuss if and how a doctor or nurse discussed with you about your cardiovascular disease risk factors	
Describe and discuss if and when you have seen the SCORE chart (show the chart)?	
Describe and discuss if and how any life-style counselling was provided to you.	
Describe and discuss your level of satisfaction with the care you received.	

In addition, clinic facility and patient pathway observations were performed in intervention clinics (*n* = 112) and control clinics (*n* = 39). During their visit in clinics the persons who collected the quantitative data from patient records also performed one patient pathway observation in each clinic where at least one physician was enrolled into the evaluation. The patient had to have an appointment because of NCD and be eligible for risk assessment according to the Ukrainian protocol (male born in 1976 or before and female born 1966 or before). The observations were performed following a protocol and a structured observation scheme including items on share of tasks between professionals, performed measurements, availability of equipment and risk assessment tools, availability and use of education material, and professionals’ performance during the visit (See Supplementary Table 3). Each observation took approximately 45 min to 1,5 h depending on the waiting time between nurses’ and doctors’ appointments.

Interviewers and data collectors were introduced to the project and the contents of the intervention and received training for the practical aspects of data collection during a two-day training. The evaluation workforce had a health background and the interviewers were already experienced in qualitative data collection.

### Analysis

We integrated the results of the different data sources through an embedded design with sequential contribution where a supplementary qualitative follow-up extension to a core quantitative data extends the initial findings by exploring how and why a particular set of results occurred [15]. Mixed-model approaches combine the strengths of qualitative and quantitative methods which enabled us to extend objectively measurable quantitative findings or care processes and outcomes with qualitative findings from interviews, emphasizing meanings and interpretation to understand professionals’ perspectives, and observations, providing an understanding of specific contexts and behaviour occurring in naturalistic settings, with a minimum intrusion by the researcher (Table 1). Preplanned linkages between these methods facilitated the integration of complementary results.

The prevalence of process and outcome indicators presented in Tables 3 and 6 were calculated separately for men and women both for intervention and control regions. Logistic regression models were fitted for control and intervention regions separately to determine age-adjusted changes in dichotomous variables between baseline and follow-up, as well as to assess age-adjusted interactions between allocation and time for all regions. A *p*-value < 0.05 was considered statistically significant.

**Table 3:** Process and outcome indicators at baseline and follow-up for women and men, % (n/N)

Characteristic^a^	Intervention				Control				Inter-action
	Baseline	Follow-up	OR (95% CI)**	*p*-value**	Baseline	Follow-up	OR (95% CI)**	*p*-value**	*p*-value**
Women									
Process indicators									
Smoking status recorded	34.0 (477/1401)	56.3 (791/1405)	2.53 (2.17–2.95)	< 0.001	0.2 (1/600)	0.2 (1/601)	–	–	–
BP measured regularly	56.1 (786/1401)	60.1 (844/1405)	1.17 (1.01–1.36)	0.037	51.2 (307/600)	56.7 (341/601)	1.24 (0.98–1.55)	0.068	0.655
ESC SCORE documented	20.0 (280/1401)	62.3 (875/1405)	6.71 (5.66–7.96)	< 0.001	0.0 (0/600)	0.0 (0/601)	–	–	–
AUDIT documented	1.1 (15/1401)	15.1 (212/1405)	17.4 (10.2–29.6)	< 0.001	0.0 (0/600)	0.0 (0/601)	–	–	–
Waist circ. Documented	9.6 (135/1401)	46.8 (657/1405)	8.31 (6.76–10.22)	< 0.001	1.7 (10/600)	1.3 (8/601)	0.76 (0.30–1.95)	0.571	< 0.001
FG documented	59.3 (831/1401)	72.0 (1011/1405)	1.75 (1.49–2.05)	< 0.001	49.2 (295/600)	53.2 (320/601)	1.15 (0.92–1.45)	0.230	0.004
TC documented	28.6 (400/1401)	56.7 (797/1405)	3.28 (2.81–3.84)	< 0.001	18.7 (112/600)	22.5 (135/601)	1.25 (0.94–1.66)	0.119	< 0.001
BMI documented	20.1 (281/1401)	66.2 (930/1405)	8.23 (6.91–9.80)	< 0.001	2.0 (12/600)	2.7 (16/601)	1.30 (0.61–2.77)	0.502	< 0.001
Outcome indicators									
BP at normal range	25.9 (275/1061)	20.7 (239/1152)	0.78 (0.63–0.95)	0.013	29.7 (130/437)	30.7 (145/472)	1.10 (0.82–1.48)	0.533	0.067
TC at normal range	29.0.0 (116/400)	30.6 (244/797)	1.08 (0.83–1.41)	0.555	30.4 (34/112)	42.2 (57/135)	1.72 (1.01–2.93)	0.047	0.145
Men									
Process indicators									
Smoking status recorded	30.6 (427/1397)	55.2 (767/1390)	2.82 (2.41–3.29)	< 0.001	0.2 (1/602)	0.2 (1/601)	–	–	–
BP measured regularly	50.9 (711/1397)	57.4 (798/1390)	1.28 (1.10–1.48)	0.002	46.5 (280/602)	55.9 (336/601)	1.41 (1.12–1.77)	0.003	0.517
ESC SCORE documented	16.5 (231/1397)	61.4 (854/1390)	8.04 (6.73–9.60)	< 0.001	0.0.0 (0/602)	0.0 (0/601)	–	–	–
AUDIT documented	1.2 (17/1397)	15.9 (221/1390)	15.6 (9.5–25.7)	< 0.001	0.0 (0/602)	0.0 (0/601)	–	–	–
Waist circ. Documented	8.7 (122/1397)	45.0 (626/1390)	8.63 (6.97–10.70)	< 0.001	2.2 (13/602)	2.3 (14/601)	0.99 (0.46–2.14)	0.980	< 0.001
FG documented	51.6 (721/1397)	66.9 (930/1390)	1.87 (1.60–2.18)	< 0.001	42.2 (254/602)	48.9 (294/601)	1.25 (0.99–1.57)	0.059	0.004
TC documented	28.1 (393/1397)	52.7 (733/1390)	2.83 (2.42–3.31)	< 0.001	16.6 (100/602)	22.3 (134/601)	1.38 (1.03–1.84)	0.031	< 0.001
BMI documented	18.7 (261/1397)	66.6 (926/1390)	8.89 (7.45–10.60)	< 0.001	2.7 (16/602)	4.0 (24/601)	1.42 (0.74–2.71)	0.292	< 0.001
Outcome indicators									
BP at normal range	32.6 (314/963)	30.9 (330/1068)	0.98 (0.80–1.18)	0.804	31.9 (123/385)	34.7 (158/455)	1.31 (0.96–1.77)	0.089	0.148
TC at normal range	38.7 (152/393)	38.7 (284/733)	1.01 (0.78–1.29)	0.970	43.0.0 (43/100)	42.5 (57/134)	0.97 (0.57–1.64)	0.901	0.951

Abbreviations: *BP* blood pressure; *ESC SCORE* cardiovascular risk; *AUDIT* Alcohol Use Disorders Identification Test; *circ*. circumference; *FG* fasting glucose; *TC* total cholesterol; *BMI* Body Mass Index; *OR* odds ratio; *CI* confidence interval

^a^Blood pressure measured regularly = Proportion of patients with two documented blood pressure measurements during the last 12 months; BP at normal range = Proportion of patients whose last two recorded blood pressure measurements were at normal range (systolic blood pressure < 140 mmHg and diastolic blood pressure < 90 mmHg), of which at least the most recent one during the last 12 months; TC at normal range = Proportion of patients whose last total cholesterol value was < 5 mmol/l

** Age adjusted

The focus group interviews were audiotaped and transcribed verbatim. The transcribed interviews were uploaded into MAXQDA qualitative data management software. Thematic analysis was used to delineate key concepts and categories from PHC providers and patients relevant to building hypotheses in relation to how the training as well as any other relevant phenomena shaped the change in PHC practices [16]. Two independent reviewers coded the transcripts, comparing initial codes and developing a codebook for subsequent coding. Inter-coder reliability was assessed following established procedures with 80% agreement or higher considered reliable. A model hypothesizing pathways in access to PHC, developed through an inductive analysis of participant narrative in combination with a priori concepts of PHC barriers and facilitators, drove the analyses of participants’ experiences. Key comparative elements in analyses included: results of the implementation, availability of developed protocols, guidelines or practice recommendations, most influential elements of the training as well as patient-related and contextual determinants. We conducted within-group and between-group comparisons, comparing PHC providers’ and patients’ perspectives with one another, among their own group, and between the groups, to tease out relational and attitudinal factors. A Qualitative Framework for Collecting and Analyzing Data in Focus Groups was used for the data analysis [17].

For the indicators evaluated in the clinic observations chi-square tests were performed to compare the categorical outcomes between the intervention and control clinics.

### Ethical review and approval

The study protocol was reviewed by the Institutional Review Board at the Ukrainian Institute on Public Health Policy. Verbal informed consent was obtained from each focus group participant and from those observed in the clinic. No personally identifiable information was collected during the interview or clinic observations.

## Results

### Detection rates for all behavioral risk factors improved

The quantitative analyses showed that recording of smoking status into the patient records almost doubled in the intervention regions over time while the activity to record smoking status was extremely low in the control regions without improvement over time (Table 3). Intervention regions also started to record harmful alcohol use and at the time of the follow-up one sixth of patients had AUDIT test results recorded to the patient records. In intervention and control clinics, recording of body mass index improved from one fifth to two thirds of patients over time, and waist circumference was measured at the time of the follow-up for almost half of the patients while at the baseline recordings were found to be only 10%.

In some regions the improvement over time was less pronounced than in others, partly because in these regions the situation was better at baseline (e.g. Vinnitsa, Kharkiv and Lviv) and thus there was less room for improvement (See Supplementary Table 4). The largest increase was observed in Poltava and Ivano-Frankivsk regions, but improvement was seen even in Lviv region where baseline performance was rather good. Recording of Body Mass Index (BMI) increased remarkably in all regions except Kyiv city (data not shown).

These findings were supported by the results observed from qualitative analyses and observation of facilities and patient pathways. Clinicians reported that they have started to pay more attention to prevention (Table 4). “Now we no longer look at one factor - for example, before we were looking only at smoking, or overweight, or … But our patients, especially of the older age, may have many different health problems! Now we are assessing cholesterol, and overweight, and smoking, and alcohol abuse... We pay attention to everything.” (Family doctor, Central region). “Well, anyway prevention is our duty. This is our main duty, and treatment is the second.” (Feldsher, Central region).

**Table 4:** Summary of content analyses – Improvements and barriers in implementing NCD prevention strategies learned at the training

Main category	Healthcare professional	Code
**Improvements**		
Organisation and working conditions	All	Focus on NCD prevention (and not only treatment) and assessment of risk factors
		General risk assessment instead of focus on single risk factors
		Access to laboratory testing improved and test were more often done
		Taking measures “in a right way”
	doctors/nurses	Improved teamwork, distribution of responsibilities (mostly, nurses do all preparatory work (measurements, tests) and doctors evaluate risk factors, and give lifestyle and treatment counseling)
		Establishing pre-medical office
		Availability of equipment on the sites
	managers	Complementary priorities (health care reform as window of opportunity for NCD prevention)
		Patient access to medicines improved
Interaction with patient	doctors/nurses/feldshers	Improved communication skills (motivational interviewing)
Emotional improvements	managers/doctors/nurses	Feeling motivated and inspired
	nurses/feldshers	Increased confidence and self-esteem due to training
**Barriers**		
Organisation and working conditions	doctors/nurses/feldshers	Lack of systematic approach to selection of the training participants (instead of training for teams of mangers, doctors, and nurses/feldshers for improved implementation)
		Lack of refresher training and possibility to share experience
		Lack of time per patient
		Workload
		Lack of management support
		Insufficient equipment on the sites
		Lack of pre-medical office
	nurses/feldshers	Lack of regular training during career
		Workload, too much paperwork
	feldshers	Poor working conditions (e.g. temperature in winter)
		Workload, working alone, too many tasks (e.g. cleaning)
		Basic equipment is missing
	managers	Lack of resources
		Competitive priorities (prevention work postponed due to health care reform)
		Lack of unified forms for monitoring project implementation (patient level and facility level)
		Lack of laboratory onsite
Interaction with patient	doctors	Lack of communication skills (motivational interviewing not applied)
		Lack of health culture in patients
	nurses/feldshers	“Patients trust the doctor more” (lifestyle counseling)
	feldshers	Social barrier to address unhealthy habits in small rural community setting
Emotional barriers	Nurses/feldshers	Feeling of inferiority, lack of trust
	feldshers	Feeling of embarrassment to discuss lifestyle with patients in their community
		Feeling overlooked/neglected (focus only on doctors and nurses)

The project also provided decision-support tools and tapes for assessment of risk factors. For example, the observation of patient pathways demonstrated that BMI tables were available in 92% of intervention clinics while only in 21% of control clinics at the time of data collection (Table 5). Also, many managers had paid attention to the equipment available. “My director ordered the tape measures... For each doctor, she ordered height meters and scales. Because, not every doctor had all that before the training.” (Family doctor, Central region).

**Table 5:** Key results from the clinic and patient pathway observations from intervention and control clinics

	Intervention	Control	Chi-Square test
	*n* = 112	*N* = 39	
**Facilities**			
Physician in same office with nurse (%)	87	100	*p* = 0.020
Scales available (%)	83	62	*p* = 0.005
Measure of height available (%)	75	66	*p* = 0.300
Measuring tape available (%)	91	89	*p* = 0.800
Blood pressure measurement equipment available (%)	99	100	NA
Different sizes of cuffs to measure blood pressure available (%)	45	54	p = 0.300
Glucometer and test strips available (%)	79	69	*p* = 0.200
Cholesterol meter and test strips available (%)	34	5	*p* < 0.001
BMI table available (%)	92	21	p < 0.001
ESC SCORE table available (%)	93	15	p < 0.001
AUDIT test available (%)	92	0	p < 0.001
Nicotine dependence test available (%)	87	0	p < 0.001
Health counselling material(s) available (%)	83	51	p < 0.001
**Performed measures**			
Height, weight and waist measurement (%)	89	79	*p* = 0.001
Blood pressure measurement (%)	100	100	NA
Random glucose (%)	86	75	*p* = 0.100
AUDIT test (%)	57	0	p < 0.001
Nicotine dependence test (%)	56	0	p < 0.001
ESC SCORE calculation (%)	81	8	p < 0.001

Abbreviations: *BMI* Body Mass Index; *ESC SCORE* Cardiovascular risk; *AUDIT* Alcohol Use Disorders Identification Test

Nurses seemed to be also more involved in assessing the behavioural risk factors. Interviewer: “Who measures the patient’s weight, height …?” “I do.” (Nurse, Western region). “Definition of body mass index. Even our patients now use this table. Patients are also interested in a healthy lifestyle, they try to prevent the onset of disease …” (Nurse, Western region). “After the training, my nurse took on more responsibilities, she became more active in this regard. Even if I am still busy with the previous patient, she invites the next one, she measures the body mass index, counts it... I would say, she performs the functions of a pre-medical office. If earlier I was not able neither force nor ask her... now she even takes the initiative!.” (Family doctor, Central region).

Also, obstacles were raised in focus groups. For example, measurement of waist circumference was a new procedure and not fully accepted: also, health professionals found it difficult to discuss alcohol use with patients. “Oh, when there are crowds of people, you do not think about waist … But if we do, I do all the measures except blood pressure.” (Nurse, Western region). “Yes, there were complaints... (laughs) Women especially reacted with some prejudice... They said: “He measured my waist! He did not have right to measure my waist! “(laughs) (Family doctor, Central region). Interviewer 1: “And if it is not visible? For example, if [female name redacted] or I come to you as patients, will you ask us about drinking alcohol?” “I don’t think so …” (Family doctor, Eastern region).

Before the intervention, both nurses and feldshers had very limited access to trainings. As this was completely new for them, they might have not benefitted from the training to the full extent. In some intervention clinics, only one professional group participated in the training and especially managers were not always participating. Especially nurses commented that the professionals should be trained as a team to get the most benefit from the trainings. “I would suggest that such trainings should be held regularly so that they become a system; at least once a year. The novel approaches approved by the Ministry of Health, the new directions in our work, we need to be informed! We would participate in such trainings with pleasure - but I think it’s better if we go together with a doctor. Then we can sit together and plan our work with our patients, and divide our duties between ourselves … To work as a team. “(Nurse, Western region).

### Assessment of total cardiovascular risk by clinicians improved

The quantitative analyses showed that the proportion of patients whose total cardiovascular risk was recorded increased from about one fifth at baseline to almost two thirds of the patients in intervention clinics while in control clinics the ESC SCORE was neither assessed at baseline nor follow-up (Table 3).

This finding was supported by the results of the qualitative assessment and observation of facilities and patient pathways. ESC SCORE tables were available in 93% of the intervention facilities (Table 5). In observation of patient pathways, the total cardiovascular risk was calculated to 81% of patients in the intervention clinics. “Prevention should be done both by the nurse and by the doctor. Cardiovascular risk - this is the first thing you need to find out in the patient! Assess risk on the SСОRE scale.” (Nurse, Western region). However, some clinicians reported that the use of SCORE tables did not become a routine. “I do not use this SCORE table. I forgot about it. Just forgot …” (Feldsher, Central region).

### Screening of biological risk factors improved

The quantitative analyses showed that the measurement and recording rates of blood pressure were already high at the baseline in intervention and control clinics (Table 3). However, a moderate and statistically significant increase was observed for both genders in intervention clinics and for men only in control clinics. A larger increase over time was observed in measurements of total cholesterol and fasting glucose in intervention clinics. In intervention regions, the proportion of patients whose total cholesterol was assessed increased statistically significantly from less than one third to over half of the patients. Fasting glucose was measured in intervention clinics at the time of follow-up from two thirds of the patients, while at the time of baseline assessment from about half of them. No improvements were observed for control clinics except for a moderate increase in total cholesterol recording in men.

This was supported by the comments from qualitative study (Table 4). Vast majority of doctors reported that they had referred their patients much more to different laboratory tests than before the intervention. “Yes, I also started recommending glycosylated hemoglobin to my patients. After the training, exactly. Because after training I understood why this matters.” (Family doctor, Eastern region). However, in addition to increased awareness on the importance of risk factor screenings, new equipment available have obviously also affected the change. Three of the intervention regions had benefitted from the simultaneously ongoing World Bank project providing some point of care testing equipment. “It is possible because we participate in a pilot on family medicine, Vinnitsa region, and we received everything we need. Every physician has a blood glucose meter and cholesterol meter in his office, and everything else. “(Family doctor, Central region). Significant differences in the proportion of the patients whose total cholesterol or blood glucose was measured were observed between regions. One explanation for that can be different possibilities for laboratory and point of care testing. “There is a blood pressure machine in my office; I also have child scales, pulse oximeter, tape measure, SCORE table – that’s all. No, there is no height meter. No glucose meter or cholesterol meter so far … And I do all the measures; my nurse did not attend the training. “(Family doctor, Western region). The existence of the WB project does not fully explain the differences as major improvement were also observed in Lviv and Ivano-Frankivsk regions and Kyiv city which did not participate in the WB project. Most likely there are also urban-rural differences in resources.

Clinicians also expressed concerns about the lack of unified system of monitoring the patients with elevated NCD risk. In some clinics separate paper forms were used, in some information was only written to patient files and some regions had electronic medical charts in use. In addition, some regions had duplicate systems (same paper and electronic forms) doubling the work load.

### Medication prescription rates improved

The quantitative analyses showed that the proportion of CVD patients prescribed a statin improved statistically significantly over time in intervention and control clinics (Table 6). Increase was also observed among diabetes patients in intervention and control clinics, but the increase was significant only among women in intervention clinics. Prescription of hypertension medication improved statistically significantly over time among all patients with hypertension and also among all patients with CVD in intervention clinics, while the improvement was only statistically significant for men in control clinics.

**Table 6:** Process and outcome indicators at baseline and follow-up for subgroups by gender, % (n/N)

Characteristic^a^	Intervention				Control				Inter-action
	Baseline	Follow-up	OR (95% CI)**	*p*-value**	Baseline	Follow-up	OR (95% CI)**	*p*-value**	*p*-value**
Women									
CVD patients									
Statin prescribed	39.1 (382/978)	54.6 (574/1051)	1.86 (1.56–2.22)	< 0.001	24.3 (78/321)	33.0 (107/324)	1.53 (1.08–2.16)	0.016	0.314
HT drug prescribed	90.3 (883/978)	93.1 (978/1051)	1.43 (1.04–1.96)	0.029	81.0 (260/321)	86.1 (279/324)	1.43 (0.94–2.19)	0.096	0.959
TC documented	33.5 (328/978)	60.2 (633/1051)	3.07 (2.56–3.69)	< 0.001	24.3 (78/321)	28.1 (91/324)	1.25 (0.87–1.78)	0.224	< 0.001
TC at normal range	30.5 (100/328)	34.4 (218/633)	1.20 (0.90–1.60)	0.215	29.5 (23/78)	48.4 (44/91)	2.18 (1.15–4.15)	0.017	0.078
HT patients									
HT drug prescribed	93.5 (1030/1102)	96.0 (1127/1174)	1.67 (1.15–2.44)	0.008	85.2 (339/398)	89.7 (366/408)	1.46 (0.95–2.23)	0.084	0.710
BP control	15.2 (134/882)	12.1 (120/995)	0.77 (0.59–1.01)	0.055	10.4 (33/316)	13.2 (46/348)	1.32 (0.82–2.12)	0.255	0.054
DM patients									
HbA1c documented	21.4 (49/229)	27.1 (71/262)	1.44 (0.95–2.20)	0.089	9.3 (5/54)	10.7 (9/84)	1.15 (0.36–3.65)	0.817	0.720
FG documented	91.7 (210/229)	92.4 (242/262)	1.16 (0.60–2.25)	0.656	77.8 (42/54)	89.3 (75/84)	2.37 (0.92–6.12)	0.076	0.231
TC documented	41.9 (96/229)	64.1 (168/262)	2.48 (1.72–3.57)	< 0.001	29.6 (16/54)	34.5 (29/84)	1.24 (0.59–2.60)	0.563	0.101
Statin prescribed	49.8 (114/229)	59.2 (155/262)	1.45 (1.01–2.08)	0.043	24.1 (13/54)	32.1 (27/84)	1.52 (0.70–3.31)	0.291	0.924
BP control	2.7 (6/226)	4.7 (12/258)	1.89 (0.70–5.14)	0.212	9.8 (5/51)	6.0 (5/84)	0.58 (0.16–2.12)	0.412	0.168
FG control	35.2 (74/210)	40.5 (98/242)	1.20 (0.82–1.77)	0.352	47.6 (20/42)	44.0 (33/75)	0.89 (0.41–1.90)	0.755	0.484
TC at normal range	29.2 (28/96)	30.4 (51/168)	1.01 (0.58–1.76)	0.979	12.5 (2/16)	37.9 (11/29)	4.36 (0.82–23.21)	0.085	0.089
Men									
CVD patients									
Statin prescribed	47.9 (395/825)	56.3 (502/891)	1.39 (1.15–1.69)	0.001	30.7 (79/257)	43.0 (120/279)	1.66 (1.16–2.37)	0.006	0.390
HT drug prescribed	87.0 (718/825)	90.1 (803/891)	1.36 (1.01–1.83)	0.046	76.3 (196/257)	84.9 (237/279)	1.68 (1.08–2.61)	0.020	0.364
TC documented	35.8 (295/825)	60.8 (542/891)	2.85 (2.34–3.47)	< 0.001	25.2 (65/257)	33.7 (94/279)	1.65 (1.12–2.42)	0.011	0.006
TC at normal range	41.0 (121/295)	43.9 (238/542)	1.12 (0.84–1.50)	0.435	49.2 (32/65)	50.0 (47/94)	1.01 (0.54–1.91)	0.969	0.793
HT patients									
HT drug prescribed	91.6 (846/924)	94.5 (924/978)	1.56 (1.09–2.24)	0.015	82.9 (271/327)	89.2 (313/351)	1.58 (1.01–2.47)	0.046	0.863
BP control	14.8 (104/705)	15.6 (126/806)	1.08 (0.82–1.44)	0.578	9.6 (24/250)	13.8 (42/305)	1.53 (0.89–2.60)	0.122	0.254
DM patients									
HbA1c documented	21.2 (33/156)	27.1 (52/192)	1.40 (0.84–2.31)	0.194	9.8 (5/51)	15.9 (11/69)	2.13 (0.67–6.80)	0.203	0.565
FG documented	83.3 (130/156)	91.1 (175/192)	2.06 (1.07–3.95)	0.030	86.3 (44/51)	79.7 (55/69)	0.65 (0.24–1.78)	0.402	0.055
TC documented	46.2 (72/156)	70.3 (135/192)	2.78 (1.78–4.32)	< 0.001	49.0 (25/51)	42.0 (29/69)	0.82 (0.39–1.73)	0.607	0.004
Statin prescribed	57.7 (90/156)	62.5 (120/192)	1.22 (0.79–1.89)	0.361	31.4 (16/51)	43.5 (30/69)	1.62 (0.75–3.48)	0.222	0.578
BP control	6.5 (10/153)	7.8 (15/192)	1.22 (0.53–2.80)	0.647	2.1 (1/47)	6.0 (4/67)	3.36 (0.35–32.02)	0.292	0.521
FG control	38.5 (50/130)	38.9 (68/175)	1.02 (0.64–1.64)	0.925	47.7 (21/44)	40.0 (22/55)	0.76 (0.34–1.69)	0.496	0.425
TC at normal range	41.7 (30/72)	34.1 (46/135)	0.72 (0.40–1.31)	0.283	36.0 (9/25)	27.6 (8/29)	0.62 (0.19–2.00)	0.420	0.873

Abbreviations: *CVD* cardiovascular disease; *HT* hypertension; *TC* total cholesterol; *BP* blood pressure; *DM* diabetes mellitus; *HbA1c* glycated haemoglobin; *FG* fasting glucose; *OR* odds ratio; *CI* confidence interval

^a^TC documented = Proportion of patients in the defined patient group with at least one documented total cholesterol measurement; Statin prescribed = Proportion of patients in the defined patient groups with stating prescribed; HT drug prescribed = Proportion of patients in the defined patient group prescribed blood pressure lowering drug; TC at normal range = Proportion of patients with cardiovascular disease whose last total cholesterol measurement was < 5 mmol/l; HT patients with BP control = Proportion of confirmed hypertensive patients whose last two blood pressure measurements were controlled (SBP < 140 mmHg and DBP < 90 mmHg), of which at least the most recent one during the last 12 months; DM patients with BP control = Proportion of diabetic patients whose last recorded blood pressure measurement was at normal range (systolic blood pressure < 130 mmHg and diastolic blood pressure < 80 mmHg); DM patients with FG control = Proportion of patients whose last fasting glucose measurement was < 7 mmol/l

** Age adjusted

Ongoing health care reform and the introduction of the national reimbursement program in 2017 obviously influenced the use of medication. This is also supported by the observation that the increase was similar also in control regions. “Available medicines – it’s such a big advantage, it has changed a lot, it is true. People who never took medicines at all, because - expensive, far to go... - now they are starting taking them, this is good. Of course, it is necessary to extend this list to other diseases, because it stimulates people to get treatment. They get prescriptions, they feel that they are taken care of, there is some attention to them. And it is free … This ensures adherence to treatment.” (Manager, Western region).

### Blood pressure, blood glucose or blood cholesterol control did not improve

The quantitative analysis showed that the proportion of hypertensive patients with good blood pressure control was rather low in intervention and control clinics and did not improve during follow-up (Table 6). Also no improvement over time was observed in control of high blood cholesterol among CVD patients or glucose among diabetes patients in intervention and control clinics in intervention and control clinics. Even though the attitudes and processes changed, the time between baseline and follow-up might have been too short to observe major changes as it needs not only change in clinical practice, but also patients’ adherence to treatments.

Detection of high values, starting medications or intensifying them is only one part of management. Patients also need to be motivated to take the medication. In the qualitative survey, all professionals complained about the lack of time as a major obstacle to thoroughly concentrate on disease management and especially in providing counselling. “Sometimes you just do not have enough time. If it was not 24 hours in a day … but at least 48, then, maybe, I would have managed all this (laughs). And if there were not 40 patients in the corridor... not 40 people in the line, but 15 or 20, as it is supposed for one shift... Then everything would be just perfect. But now we have … I personally have 40 people for appointments per shift.” (Family doctor, Central region). “You know, theу were so vivid, these activities and exercises... We were traveling home with my doctor, discussing: “It should be done in this way, it should be done in that way …” We were so inspired! Well, then we came home... And somehow … we come to naught... Because we just have no time, no time to do this... Well, of course we have implemented something, but not everything. Because it is really not enough - 15 min per patient...” (Nurse, Central region).

In the qualitative research, it was observed that the professionals found motivational interviewing as a new method and thus not so easily adjustable to routine practice. “I would also like to learn some psychological moments, how to find an approach and communicate with a patient, some tricks... How to make a patient do what is needed, how to talk with him properly, so that he will hear and believe you …” (Family doctor, Eastern region). However, the patients had noticed changes in treatment culture. “Before, it was a little bit different, they mostly prescribed medication. Now more attention is paid to prevention and preventive examinations. The doctor explains everything in a lay language, so that the patient understands that he himself needs it, and not the doctor only. This is our health, and this is not only doctor’s responsibility, but also our responsibility...” (Female patient, Central region). “I liked very much that the doctor – well, not only those medicines … She said that I need to cut salt consumption, I should consume less fat to keep healthy diet … And you know, I have lost 8 kilos within past year!” (Female patient, Central region). Also the observation of facilities and patient pathways supported this finding as much more preventive counselling was provided to patients in intervention areas compared with control areas. In observations, 96% of patients got some kind of preventive or health promoting counselling either by a physician or a nurse in intervention regions, compared with only 41% in control regions.

## Discussion

### Summary of findings

This study demonstrates the effects of a capacity building intervention among primary health care professionals carried out in Ukraine using mixed-methods evaluation. Quantitative data was collected from patient records. Such approach using real world data (RWD) to generate real world evidence (RWE) is useful for assessing effectiveness of interventions that are not well suited to being tested in RCTs (for example health service level interventions) [18].

Some improvement was seen in detection rates for behavioural and biological risk factors – many of which were significant only in intervention clinics. Improvement was also observed in medication prescription rates, but those were very similar also in control clinics. Improvements in control of blood pressure, blood glucose and blood cholesterol were not seen during the implementation period evaluated.

The findings from the qualitative data supported the changes observed in quantitative quality of care indicators. Trainings addressing the importance of total risk assessment, provision of tools and equipment and giving more responsibility to nurses had increased the interest and capacity to actively identify risk factors and to record those. The new national reimbursement programme for essential medicines [19] seemed to be the key driver for improved prescription rates of hypertension medication and statins. This also explains the similarities between intervention and control clinics in that regard. According to qualitative findings, even though improved communication skills were reported, many factors such as lack of time per patient and uncertainty in using newly learned motivational interviewing techniques can be factors that explain modest or no improvement in the control of risk factors in certain settings. Most patients had however observed change in treatment cultures towards a more preventive approach.

### Interpretation of findings

Changing clinical practice in health service delivery is a complex task with unique considerations based on geographical, political, economic, and historical contexts. Known challenges to implementing essential interventions for CVD risk management in Eastern Europe includes inconsistent international guidance, lack of national capacity for evidence-based healthcare, limited access to essential medicines and technologies, inconsistent national guidelines, and limited experience in guideline implementation and clinical epidemiology [20, 21]. Many of these known challenges were addressed through the larger SDC project including prioritizing NCDs at a national level, identifying and mapping existing resources, and engaging key stakeholders. The study herein focused more specifically on tailoring interventions to the Ukrainian health system and generating local evidence for the purpose of quality improvement and mainstreaming. All of these activities are known to help implement essential NCD interventions in settings similar to Ukraine [22].

The Consolidated Framework for Implementation Research (CFIR), which outlines factors critical to successful implementation, has been previously described in relation to CVD risk management in Eastern European and Central Asian Countries [22, 23]. The explanatory qualitative findings of the present study mostly relate to the inner setting – the primary health care centers themselves – rather than the intervention characteristics, characteristics of individuals, the outer setting, or overall process. The most prominent domains of the inner setting, as defined by the CFIR, in the qualitative results were: implementation climate, tension for change, compatibility, relative priority, readiness for implementation, leadership engagement, available resources, and access to knowledge and information [22, 23]..

Changes quite inevitably meet resistance. Some factors leading to resistance are stability, established structures, traditional hierarchies, institutional climates and existing roles and competencies of professionals [24–26]. In terms of the implementation climate in primary health care, or the capacity for change, not all the managers in the participating clinics participated themselves in the trainings, which most likely in addition to their existing skills in leading organization change affected their commitment for implementation of the new interventions [27]. Furthermore, clinicians were inspired by the training and ready to change their practices, but when returning back to offices were overwhelmed with time constraints and established structures prevented them from reorganizing their practices or the content of their work. Coupled together with lack of support and leadership engagement needed for uptake of new processes [28], these findings suggest an insufficient tension for change within many primary health care centers. Some centers, however, showed a greater degree of readiness for implementation with a high degree of compatibility with the intervention package, as demonstrated by the establishment of “pre-medical offices” to increase the capacity to conduct CVD risk assessment and enhance the roles of nurses in the management of NCDs. In the process of changing clinical practice in health service delivery the successes and failures are greatly determined by people and processes either enabling or resisting the change [24]. Successful change is dependent on how people change their work [29].

While clinicians found the training useful, the qualitative findings also demonstrated that the relative priority for implementation was generally low (due to competing demands and lack of managerial support) and the complexity of the intervention was high after one training only. While initially the access to knowledge and information was good through the training, participants specifically highlighted the need for continued medical education to aid in implementation in their own medical practices.

On this point in particular, there is a vast evidence base examining the impact of continuing medical education (CME) on physician behavior and patient outcomes. An overview of 39 systematic reviews [30] parsed the evidence and highlighted consistent improvements in clinician knowledge and patient outcomes as a result of CME. While our results demonstrated changes in physician behavior, it did not show a change in patient outcomes. While the initial training provided was based on a needs assessment and used interactive learning methods, both known to improve quality of care, it was lacking in continuity which is an important aspect of maximizing quality improvement from CME. In future, greater emphasis on continuity (e.g. follow up support and training) and individual or practice level needs assessments may help increase the impact on patient outcomes.

### Strengths and limitations

WHO was carrying a much broader piece of work to improve NCD prevention and health promotion during the period of this study (2016–2019). This included initiatives on NCD policy development, such as the development and approval of a National NCD Action Plan till 2030, NCD prevention and health promotion in school settings where school nurses and local community were involved, as well as social marketing campaigns for alcohol control and a communication strategy on NCD prevention. We cannot be sure to what extent these broader initiatives impacted on the knowledge or behavior of clinicians and patients within our study. Nevertheless, we would have expected such initiatives to impact on both pilot and control clinics equally.

As mentioned in the introduction, broader health systems strengthening efforts were underway in Ukraine during the period that this project was implemented. The Affordable Medicines Programme was introduced in Ukraine in April 2017 to provide patients with improved access to 23 outpatient medicines for the treatment of chronic noncommunicable diseases [19]. Its evaluation confirmed that the Programme contributed to a significant increase in access to needed outpatient medicines in Ukraine although uptake across regions was uneven. As the prescription rates improved also in control clinics the effect observed is most likely due to the Affordable Medicines Programme and the independent effect of training cannot be analysed.

One of the limitations is a relatively short implementation period (1.5 to 2 years) as the retrospective evaluation happened quite soon after the training had been finished. Adaptation of new practices takes time, so some of the benefits of the training and the obtained new skills might not become visible in such a short time. Changes in professionals’ performance during the pathway observations cannot be fully ruled out. However, we assume the effect to be small as the timing of the observation was not known beforehand and the changes in performance compared with usual practice caused by the observer are likely to be similar in intervention and control clinics. While the use of control regions as comparisons was helpful in contextualizing and determining the significance of the changes observed in pilot regions, this method is less than ideal since it does not allow for removal of confounding factors that would be achieved through randomization.

Patient records are quite an objective data source and give a good understanding of care processes and outcomes as they avoid non-response bias. However, some information might be missing if for example measurements done during the appointment are not adequately recorded. Information on socioeconomic factors, diet, and physical activity are usually lacking.

## Conclusions

This large-scale capacity building intervention improved professionals’ knowledge, skills and possibilities in detecting and assessing the risk and risk factors of non-communicable diseases. The participants reported paying more attention to prevention after the training. Changes were also seen in clinical practice. However, the intervention was not enough to create improvements in treatment outcomes at least not in such a short period. In addition to educating professionals, providing some tools and materials and providing support and guidance to health authorities, the improvement of quality of care would need more comprehensive systems change [31]. Including the managers in the training, and training teams of professionals together, as well as follow-up training for health workers might have enhanced impact. Broader changes would also need to overcome the challenges associated with lack of time available in consultations.

## Supplementary Information

**Additional file 1: Supplementary Table 1.** Contents of the online standardized data collection form used to extract data from individual patient records. **Supplementary Table 2.** The semi-structured focus group interview scheme. **Supplementary Table 3.** Clinic facility and patient pathway observation scheme. **Supplementary Table 4.** Process and outcome indicators at baseline and follow-up by region, % (n).

## Data Availability

The datasets generated and/or analysed during the current study are not publicly available due to security requirements of health data but are available from the corresponding author on reasonable request.

## Abbreviations

BMI: Body Mass Index
CFIR: Consolidated Framework for Implementation Research
CME: Continuing medical education
CVD: Cardiovascular diseases
ESC SCORE: European Society of Cardiology Cardiovascular risk score
PHC: Primary Health Care
NCD: Non-communicable disease
RWD: Real World Data
RWE: Real World Evidence
WHO: World Health Organization
