# Supplementary material for: Capacity building of health care professionals to perform interprofessional management of non-communicable diseases in primary care – experiences from Ukraine
Source: BMC Health Serv Res. 2021 Jan 27;21:91. doi: 10.1186/s12913-021-06068-1 (PMC7839221; doi:10.1186/s12913-021-06068-1)
Supplement: Supplementary file 1 — Additional file 1: Supplementary Table 1. Contents of the online standardized data collection form used to extract data from individual patient records. Supplementary Table 2. The semi-structured focus group interview scheme. Supplementary Table 3. Clinic facility and patient pathway observation scheme. Supplementary Table 4. Process and outcome indicators at baseline and follow-up by region, % (n). [file 12913_2021_6068_MOESM1_ESM.docx]

Supplementary Table 1. Contents of the online standardized data collection form used to extract data from individual patient records

| Data collector: name/code |
| --- |
| Date of Data Extraction |
| Write the Clinic Name |
| Write the name/code of the doctor whose patient this client is |
| Data collection period (2018 or 2016) |
| Date of Birth (MM-DD-YYYY) |
| Sex (M/F) |
| Smoking Status (Y/N) |
| Diagnosis of Hypertension (Y/N) |
| Date of Hypertension Diagnosis (MM-DD-YYYY) |
| Can you find one or more blood pressure readings? (Y/N) |
| Most Recent Systolic Blood Pressure (mmHg) |
| Most Recent Diastolic Blood Pressure (mmHg) |
| Date of the Most Recent Blood Pressure Measurement (MM-DD-YYYY) |
| Can you find a second most recent blood pressure reading? (Y/N) |
| Second most recent systolic blood pressure (mmHg) |
| Second most recent diastolic blood pressure (mmHg) |
| Date of the second most recent blood pressure (MM-DD-YYYY) |
| Diagnosis of Diabetes (Type 1, Type 2, No) |
| Can you find one or more HbA1c measurements? (Y/N) |
| Most recent HbA1c reading (%) |
| Date of the most recent HbA1c measurement? (MM-DD-YYYY) |
| Can you find another HbA1c measurement? (Y/N) |
| Second most recent HbA1c reading |
| Date of the second most recent HbA1c reading? (MM-DD-YYYY) |
| Can you find one or more fasting glucose measurements? (Y/N) |
| Most recent fasting glucose reading (mmol/L |
| Date of the most recent fasting glucose measurement? (MM-DD-YYYY) |
| Can you find another fasting glucose measurement? (Y/N) |
| Second most recent fasting glucose reading (mmol/L) |
| Date of the second most recent fasting glucose reading? (MM-DD-YYYY) |
| Can you find one or more total cholesterol measurements? (Y/N) |
| Most recent total cholesterol reading (mmol/L) |
| Date of the most recent total cholesterol reading (MM-DD-YYYY) |
| Can you find another total cholesterol measurement? (Y/N) |
| Second most recent total cholesterol reading (mmol/L) |
| Date of the second most total recent cholesterol reading (MM-DD-YYYY) |
| Can you find one or more LDL measurements? (Y/N) |
| Most recent LDL reading (mmol/L) |
| Date of the most recent LDL reading (MM-DD-YYYY) |
| Can you find another LDL measurement? (Y/N) |
| Second most recent LDL reading (mmol/L) |
| Date of the second most recent LDL reading (MM-DD-YYYY) |
| Was the patient prescribed a statin? (Y/N) |
| What was the date of the prescription? (MM-DD-YYYY) |
| What was the name of the (statin) drug? |
| What was the dose of the (statin) drug? (mg) |
| Does the patient have existing CVD? (Y/N) |
| State the type of CVD |
| Has the patient been prescribed Aspirin? (Y/N) |
| What was the most recent date that aspirin was prescribed? (MM-DD-YYYY) |
| Has the patient been prescribed any blood pressure-lowering medication? (Y/N) |
| What was the most recent date that blood pressure-lowering medications were prescribed? (MM-DD-YYYY) |
| Can you find a documented ESC SCORE risk score? (Y/N) |
| Enter the most recent documented ESC SCORE risk score (%) |
| What was the date the risk score was documented? (MM-DD-YYYY) |
| Can you find a documented BMI? (Y/N) |
| Enter the most recent documented BMI (kg/m2) |
| What was the date the BMI was documented? (MM-DD-YYYY) |
| Can you find a documented waist circumference measure? (Y/N) |
| Enter the most recent documented waist circumference (cm) |
| What was the date the waist circumference was documented? (MM-DD-YYYY) |
| Can you find a documented AUDIT score? (Y/N) |
| Enter the most recent documented AUDIT score |
| What was the date the AUDIT score was documented? (MM-DD-YYYY) |
| Can you find a documented result of nicotine dependence test? (Y/N/NA) (if the person is not a smoker put NA) |
| Enter the most recent documented results of the nicotine dependence test. |
| What was the date the nicotine dependence test was documented? (MM-DD-YYYY) |
| Please record any important notes about the data extraction here. Examples include an error you think may have been made, clarification of the units for measurements (e.g. mmol/L vs mg/dL). Or notes that you would like for yourself. |

Supplementary Table 2. The semi-structured focus group interview scheme

| **Clinic managers** | |
| --- | --- |
| Describe and discuss the role that managers played in implementing the project. | - What were the main challenges to implementation? - Discuss if or how organization of care needed to be changed. Was it possible? Why or why not? - Discuss if or how division of tasks (tasking sharing) need to be changed. Was it possible? Why or why not? - Discuss if or how the workforce (doctors, nurses, feldshers, administrators) needed additional training. Was it possible? Why or why not? - Discuss if or how equipment and materials needed to be changed. Was it possible? Why or why not? - Discuss if or how access to laboratory testing needed to be changed. Was it possible? Why or why not? - Discuss if or how access to medicines needed to be changed. Was it possible? Why or why not? |
| Describe and discuss the results or outcomes of the implementation. | - Specifically, what were the outcomes you achieved? And how do you know they were achieved? - Discuss how you monitored how changes in practice changed patient outcomes. If you didn’t, why not? - Describe if and how clinical auditing was used in the clinic. If it isn’t used, why not? |
| Describe and discuss how the role of nurses and/or feldshers changed? | - If it did not, why not? |
| Describe and discuss how local protocols, guidelines, or practice recommendations were developed after training to support clinical decision making. | - If this wasn’t done, why not? - If it this was done, were they used? |
| Describe and discuss how patients are contacted or reminded to come to their follow-up medical appointment (“recall systems”) | - How were they developed? - Were they used? - What proportion of patients would show up to their appointment when requested? |
| Describe and discuss any recommendations you would give to other clinics who want to implement the project. |  |
| **Doctors** | |
| Describe and discuss what you learned from the training workshop | - How did it influence your practice? - How did it impact the quality of care you provide? How do you know? - How did this changed the care you provide with respect to primary prevention and early detection of NCDs (including treatment and monitoring)? |
| Describe and discuss if and how the division of tasks of doctors, feldshers and nurses changed after the training? | - Please answer specifically for each of the following tasks: o Total risk assessment o Lifestyle counselling o Motivational interviewing o Risk factor measurement (e.g. blood pressure, AUDIT, BMI) |
| Describe and discuss the support you had from your clinic (e.g. managers) to make implement the changes you learned in the training | - How did they support you? - How did they complicate or make it more difficult? |
| **Feldshers** | |
| Describe and discuss what you learned from the training workshop | - How did it influence your practice? - How did it impact the quality of care you provide? How do you know? - How did this changed the care you provide with respect to primary prevention and early detection of NCDs (including treatment and monitoring)? |
| Describe and discuss if and how the division of tasks of doctors, feldshers and nurses changed after the training? | - Please answer specifically for each of the following tasks: o Total risk assessment o Lifestyle counselling o Motivational interviewing o Risk factor measurement (e.g. blood pressure, AUDIT, BMI) |
| Describe and discuss the support you had from your clinic (e.g. managers) to make implement the changes you learned in the training | - How did they support you? - How did they complicate or make it more difficult? |
| Describe and discuss how your confidence in dealing with patients changed? | - How has it changed? - What are the benefits and downsides of these changes? |
| **Nurses** | |
| Describe and discuss what you learned from the training workshop | - How did it influence your practice? - How did it impact the quality of care you provide? How do you know? - How did this changed the care you provide with respect to primary prevention and early detection of NCDs (including treatment and monitoring)? |
| Describe and discuss if and how the division of tasks of doctors, feldshers and nurses changed after the training? | - Please answer specifically for each of the following tasks: o Total risk assessment o Lifestyle counselling o Motivational interviewing o Risk factor measurement (e.g. blood pressure, AUDIT, BMI) |
| Describe and discuss the support you had from your clinic (e.g. managers) to make implement the changes you learned in the training | - How did they support you? - How did they complicate or make it more difficult? |
| Describe and discuss how your confidence in dealing with patients changed? | - How has it changed? - What are the benefits and downsides of these changes? |
| **Patients** | |
| Describe and discuss any changes in the care you received during the last year | - Changes in who gave you care? - Changes in treatment provided? |
| Describe and discuss how your blood pressure, blood sugar, waist circumference, and/or weight have been measured | - Who measured these? - How often were they measured? |
| Describe and discuss if and how you have been asked about your smoking, alcohol use, diet, physical activity? |  |
| Describe and discuss if and how a doctor or nurse discussed with you about your cardiovascular disease risk factors |  |
| Describe and discuss if and when you have seen the SCORE chart (show the chart)? | - What is a cardiovascular risk score? - How was the meaning of the risk score explained to you? - Describe how cardiovascular risk scores affect your decisions about health and treatment |
| Describe and discuss if and how any life-style counselling was provided to you. | - What about self-care counselling? - Describe the advice and counselling and why you did or didn’t think it was clear? |
| Describe and discuss your level of satisfaction with the care you received. | - How would you rate the quality of the care you received on a scale of 1-10 (1= worst 10=best) and why? - Do you feel that you have an opportunity to get all of your questions about your health answered through your health care? |

Supplementary Table 3. Clinic facility and patient pathway observation scheme

| **Observation scheme for patient pathway** | | | | | |
| --- | --- | --- | --- | --- | --- |
| Is the nurse/feldsher operating in the same room with the physician | | | YES/NO | | Notes |
| Are the following equipment/material available in the appointment room? (N=nurse has, F=feldsher has, D=physician has, NO=no one has; several can be selected) | | | | | |
|  | Scale | | N/F/D/NO | |  |
|  | Measure of height | | N/F/D/NO | |  |
|  | Waist measurement tape | | N/F/D/NO | |  |
|  | Blood pressure measurement equipment | | N/F/D/NO | |  |
|  | Different size of cuffs to measure blood pressure | | N/F/D/NO | |  |
|  | Glucometer and test strips | | N/F/D/NO | |  |
|  | Cholesterometer and test strips | | N/F/D/NO | |  |
|  | BMI table | | N/F/D/NO | |  |
|  | SCORE table | | N/F/D/NO | |  |
|  | AUDIT tool | | N/F/D/NO | |  |
|  | Nicotine dependence test table | | N/F/D/NO | |  |
|  | Health counselling material(s) | | N/F/D/NO | |  |
| Division of tasks between nurses/feldshers and physicians | | |  | | |
|  | Who performs height, weight and waist circumference measurements (N=nurse, F=feldsher, D=physician, NO=no one did the measurements) | | N/F/D/NO | |  |
|  | Who performs blood pressure measurement | | N/F/D/NO | |  |
|  | Who performs random glucose measurement | | N/F/D/NO | |  |
|  | Who performs the AUDIT test | | N/F/D/NO | |  |
|  | Who performs the nicotine dependence test (N=nurse, F=feldsher, D=physician, NO=no one did the measurements, NA=patient is not a smoker) | | N/F/D/NO/NA | |  |
|  | Who is calculating the SCORE | | N/F/D/NO | |  |
| Is preventive/health promoting counselling provided during the visit (any counselling regarding smoking cessation, weight management, reduction of alcohol use, physical activity, healthy nutrition)? | | | Y/N | |  |
|  | Who provides the health counselling (N=nurse, F=feldsher, D=physician, BO=both nurse/feldsher/physician, NO=no one did the counselling) | | N/F/D/BO/NO | |  |
| Is self-care counselling provided during the visit (any counselling regarding self-care of diabetes or hypertension e.g. use of medication)? | | | Y/N | |  |
|  | Who provides the health counselling (N=nurse, F=feldsher, D=physician, BO=both nurse/feldsher/physician, NO=no one did the counselling) | | N/F/D/BO/NO | |  |
| Does the nurse/feldsher ensure that the patient understands the advice given? (e.g. by asking directly whether the patient has understood the advice, repeating the advice, providing the patient a possibility to ask more, summarizing the key point) | | | Y/N | |  |
| Is the blood pressure measurement technique of the person performing the measurement correct? | | | Y/N | |  |
| **General observations in the clinic** | | | | | |
| Is there health promotion material available in the clinic (e.g. posters on the walls, leaflets for patient to read or take with them etc.) | | none/only few/some, but not covering well the key health topics/ a lot covering well the key health topics | | Notes/explanations | |
| Is there possibility to draw blood in the clinic? | | Y/N | |  | |
| Is there a lab in the clinic which allows to perform the following tests: - Glucose - Cholesterol and its fractions - Creatinine - Other relevant tests – provide explanations | | Y/N Y/N Y/N Y/N | |  | |
| How many nurses there are per physician in the clinic? | |  | |  | |

Supplementary Table 4. Process and outcome indicators at baseline and follow-up by region, % (n)

| **Characteristic** | | **Baseline** | **Follow-up** |
| --- | --- | --- | --- |
| **Intervention** | |  |  |
| Vinnytska | | 100 (401) | 100.0 (402) |
|  | Smoking status recorded | 62.1 (249) | 68.9 (277) |
|  | BP measured regularly | 71.8 (288) | 75.6 (304) |
|  | ESC SCORE documented | 8.5 (34) | 52.2 (210) |
|  | AUDIT documented | 0 (0) | 0 (0) |
|  | Waist circ. documented | 16 (64) | 70.6 (284) |
|  | FG documented | 75.8 (304) | 91 (366) |
|  | TC documented | 36.2 (145) | 63.4 (255) |
|  | BMI documented | 31.9 (128) | 89.1 (358) |
| Dnipropetrovska | | 100.0 (405) | 100.0 (402) |
|  | Smoking status recorded | 8.6 (35) | 10.7 (43) |
|  | BP measured regularly | 59.3 (240) | 64.2 (258) |
|  | ESC SCORE documented | 47.4 (192) | 59.7 (240) |
|  | AUDIT documented | 0 (0) | 0.2 (1) |
|  | Waist circ. documented | 25.4 (103) | 39.3 (158) |
|  | FG documented | 49.4 (200) | 56.5 (227) |
|  | TC documented | 21.5 (87) | 31.1 (125) |
|  | BMI documented | 36 (146) | 57.5 (231) |
| Ivano-Frankivska | | 100.0 (403) | 100.0 (402) |
|  | Smoking status recorded | 7.2 (29) | 59.5 (239) |
|  | BP measured regularly | 33.7 (136) | 39.3 (158) |
|  | ESC SCORE documented | 4.7 (19) | 88.6 (356) |
|  | AUDIT documented | 0 (0) | 30.3 (122) |
|  | Waist circ. documented | 2.5 (10) | 69.2 (278) |
|  | FG documented | 57.8 (233) | 77.9 (313) |
|  | TC documented | 26.3 (106) | 89.3 (359) |
|  | BMI documented | 10.9 (44) | 84.3 (339) |
| Kyiv city | | 100.0 (400) | 100.0 (403) |
|  | Smoking status recorded | 1 (4) | 2.7 (11) |
|  | BP measured regularly | 45.5 (182) | 46.7 (188) |
|  | ESC SCORE documented | 0.8 (3) | 2 (8) |
|  | AUDIT documented | 0 (0) | 0 (0) |
|  | Waist circ. documented | 7.5 (30) | 11.7 (47) |
|  | FG documented | 33.8 (135) | 46.7 (188) |
|  | TC documented | 21.5 (86) | 33.3 (134) |
|  | BMI documented | 17.3 (69) | 24.1 (97) |
| Lvivska | | 100.0 (385) | 100.0 (379) |
|  | Smoking status recorded | 43.4 (167) | 77 (292) |
|  | BP measured regularly | 46.8 (180) | 54.6 (207) |
|  | ESC SCORE documented | 0.5 (2) | 74.4 (282) |
|  | AUDIT documented | 0 (0) | 0.3 (1) |
|  | Waist circ. documented | 0.3 (1) | 20.6 (78) |
|  | FG documented | 31.9 (123) | 46.2 (175) |
|  | TC documented | 16.9 (65) | 55.1 (209) |
|  | BMI documented | 13 (50) | 66 (250) |
| Poltavska | | 100.0 (400) | 100.0 (401) |
|  | Smoking status recorded | 22.3 (89) | 81.5 (327) |
|  | BP measured regularly | 54 (216) | 62.6 (251) |
|  | ESC SCORE documented | 42.8 (171) | 88.5 (355) |
|  | AUDIT documented | 8 (32) | 62.1 (249) |
|  | Waist circ. documented | 12.3 (49) | 84.8 (340) |
|  | FG documented | 56.5 (226) | 77.6 (311) |
|  | TC documented | 37.8 (151) | 62.3 (250) |
|  | BMI documented | 21.3 (85) | 87.5 (351) |
| Kharkivska | | 100.0 (404) | 100.0 (406) |
|  | Smoking status recorded | 81.9 (331) | 90.9 (369) |
|  | BP measured regularly | 63.1 (255) | 68 (276) |
|  | ESC SCORE documented | 22.3 (90) | 68.5 (278) |
|  | AUDIT documented | 0 (0) | 14.8 (60) |
|  | Waist circ. documented | 0 (0) | 24.1 (98) |
|  | FG documented | 81.9 (331) | 88.9 (361) |
|  | TC documented | 37.9 (153) | 48.8 (198) |
|  | BMI documented | 5 (20) | 56.7 (230) |
| **Control** | |  |  |
| Sumska | | 100. 0 (400) | 100.0 (401) |
|  | Smoking status recorded | 0.3 (1) | 0 (0) |
|  | BP measured regularly | 61 (244) | 72.1 (289) |
|  | ESC SCORE documented | 0 (0) | 0 (0) |
|  | AUDIT documented | 0 (0) | 0 (0) |
|  | Waist circ. documented | 0 (0) | 0.5 (2) |
|  | FG documented | 54 (216) | 66.8 (268) |
|  | TC documented | 14.8 (59) | 26.2 (105) |
|  | BMI documented | 0 (0) | 3.7 (15) |
| Ternopilska | | 100.0 (400) | 100.0 (400) |
|  | Smoking status recorded | 0 (0) | 0.3 (1) |
|  | BP measured regularly | 40.5 (162) | 38.5 (154) |
|  | ESC SCORE documented | 0 (0) | 0 (0) |
|  | AUDIT documented | 0 (0) | 0 (0) |
|  | Waist circ. documented | 0 (0) | 0 (0) |
|  | FG documented | 34.5 (138) | 37 (148) |
|  | TC documented | 12 (48) | 11.5 (46) |
|  | BMI documented | 0 (0) | 0 (0) |
| Cherkaska | | 100.0 (402) | 100.0 (401) |
|  | Smoking status recorded | 0.2 (1) | 0.2 (1) |
|  | BP measured regularly | 45 (181) | 58.4 (234) |
|  | ESC SCORE documented | 0 (0) | 0 (0) |
|  | AUDIT documented | 0 (0) | 0 (0) |
|  | Waist circ. documented | 5.7 (23) | 5 (20) |
|  | FG documented | 48.5 (195) | 49.4 (198) |
|  | TC documented | 26.1 (105) | 29.4 (118) |
|  | BMI documented | 7 (28) | 6.2 (25) |
| Abbreviations: BP, blood pressure; ESC SCORE, cardiovascular risk; AUDIT, Alcohol Use Disorders Identification Test; circ., circumference; FG, fasting glucose; TC, total cholesterol; BMI, Body Mass Index. | | | |
